# Tribological Behavior of TiC Particles Reinforced 316Lss Composite Fabricated Using Selective Laser Melting

**DOI:** 10.3390/ma12060950

**Published:** 2019-03-21

**Authors:** Jing Li, Zhanyong Zhao, Peikang Bai, Hongqiao Qu, Minjie Liang, Haihong Liao, Liyun Wu, Pengchen Huo

**Affiliations:** 1 School of Materials Science and Engineering, North University of China, Taiyuan 030051, China; jing.li3d@hotmail.com (J.L.); 15035691367@163.com (H.Q.); lmjnwpu@hotmail.com (M.L.); lhh@nuc.edu.cn (H.L.); wuliyunnuc@126.com (L.W.); nuchpc@126.com (P.H.); 2 Shanxi Institute of Technology, Yangquan 045000, China

**Keywords:** 316Lss matrix composite, selective laser melting, TiC particles, tribological behavior

## Abstract

In order to improve the abrasion performance of 316Lss, make full use of its advantages and broaden its application fields, the tribological behavior of the TiC particles reinforced 316Lss composites—which were manufactured by selective laser melting (SLM)—were investigated. In this study, GCr15 bearing steel was selected as the friction material and experiments on the sliding friction and wear under different loads of 15 N, 25 N and 35 N at the sliding speeds of 60, 80 and 100 mm/min were carried out, respectively. The results show that the wear performance of the TiC/316Lss composite is higher than that of the matrix during the friction and wear experiments under all conditions and the wear rate of the TiC/316Lss composite decreases with increasing the friction rate. Similar to the wear mechanism under different loads, it changes from abrasive wear to delamination wear and severe oxidative wear. At the same time, the mechanical mixed layer formed at a high speed has a protective effect on the matrix. The reason for this phenomenon is that the mechanical properties of the TiC/316Lss composites are significantly improved due to the addition of TiC particles, the refinement of cells near the TiC particles and the formation of a large number of dislocations. In addition, due to the presence of the TiC particles, the hardness and strength of the TiC/316Lss composites are greatly improved, thus the processing hardening ability of sub-surface has been improved.

## 1. Introduction

As one of the additive manufacturing technologies, selective laser melting (SLM) technology can realise the rapid formation of complex structural parts, greatly reducing processing procedures and reducing the development costs and risks. At present, it has been widely used in biomedical parts, radiator parts, ultra-light structural parts and micro-devices, as well as in other fields [1,2,3]. As a widely used structural material, 316L austenitics has attracted wide attention in the aviation, nuclear power plant, biomedicine and petrochemical industries due to its good plasticity, toughness, weldability and good corrosion resistance in oxidizing and reducing media [4,5]. However, 316Lss has relatively low strength and poor abrasion performance compared with other alloy steels, such as martensitic steels, which limits its further applications [6,7,8,9]. Therefore, it is necessary to improve the friction and wear properties of 316Lss and study its wear mechanism. In recent years, in order to significantly improve the mechanical properties and tribological behavior of metal materials, an important research direction has been ceramic particle reinforced 316Lss. Ceramic particle reinforced 316Lss matrix composites with low density, high strength and high stiffness are added to this matrix, which can improve abrasion performance and high temperature mechanical properties while reducing material density [10,11].

Some researchers have already reported on the mechanical properties of ceramic particle reinforced metal matrix composites. Xu et al. [12] studied that by comparing with the pure Inconel 625 coatings, the microhardness and tensile strength of the TiC reinforced Inconel 625 coatings are significantly improved. Guo et al. [13] investigated that the hardness increased from 219 to 1056 HV0.2 of the FSed 316L/TiB2 coating, because of the presence of TiB2 and the intermetallic phase, the austenite cells are finer, and the orientation density is higher. Zhang et al. [14] found that by adding TiB2 particles, the density of Inconel 625/TiB2 composites formed by the formation of pores and cracks can be reduced. Almangour et al. [15] found that the TiB2/316L nanocomposites exhibit higher microhardness than SLM treated 316Lss samples, while exhibiting lower friction and wear rates but when adding the 15 vol.% TiB2, the TiB2/316L composite showed significant abrasion performance although there are some porosities. Besides, TiC reinforced 316Lss matrix composites attract great attention due to its advantages such as clean interface and nanoscale reinforcements formed controllably [16,17]. What is more, TiC ceramic particles have been widely used as reinforcements in metal matrix composites due to lower density, higher hardness, excellent corrosion and wear resistance [18]. However, previous research on the tribological behaviors of composites mainly focused on micron particle reinforced composites. Until now, there have been few publications on the tribological behavior of SLM-formed TiC/316Lss composites. Therefore, it is essential to study the tribological behavior of the TiC/316Lss composites to clarify the wear mechanism under various wear conditions. 

In this study, the friction and wear properties of the TiC/316Lss composite are studied. The influence of the subsurface structure evolution and the addition of the TiC particles on the friction and wear properties of the 316Lss matrix was analyzed.

## 2. Experimental Procedure

### 2.1. Manufacture of Composite Materials and SLM Process

**Figure 1:**
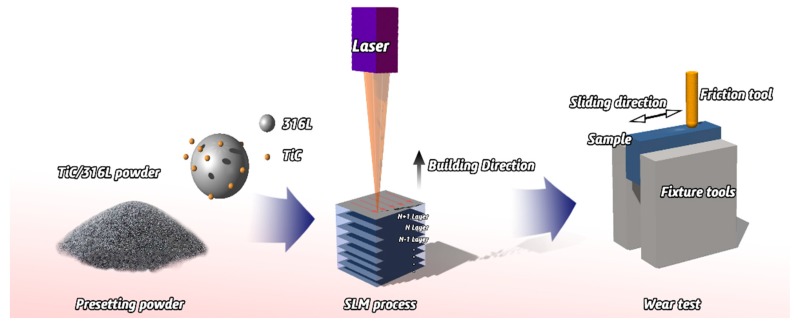
Schematic diagram of experimental process.

In this study, experiments were carried out in an argon atmosphere with 316Lss spherical powder with a size of 45 μm and the average particle size of TiC powder is 2-5 μm. A Pulverisette 4-ball mill (Fritsch GmbH, Idar-Oberstein, Germany) machine is used to prepare the composite powder of 2 wt% TiC; the SLM system (Renishaw AM 250, Renishaw, Gloucestershire, England) was used to manufacture the samples and the parameters were fixed as follows: the focused beam diameter is 70 μm, the scanning speed is 1200 mm/s, the laser power is 200 W, the hatch spacing is 60 μm and the layer thickness is 30 μm. The 10 mm × 10 mm × 10 mm cube specimens were produced as samples (Figure 1).

### 2.2. Mechanical Properties Test

The microhardness of the samples were measured with a Vickers micro-hardness tester (TMHVS-1000, Shanghai Tuming Optical Instrument CO., LTD, Shanghai, China) under a 200 g load and lasted for 15 s. Similarly, the average of 10 hardness values was taken as the final experimental value and three samples of each material were tested.

### 2.3. Friction and Wear Test

The reciprocating friction and wear test for the 316Lss and TiC/316Lss composite were conducted under the different sliding speeds of 60, 80 and 100 mm/min at the loads of 15 N, 25 N and 35 N respectively, at room temperature. GCr15 bearing steel was selected as the friction material. The duration of the test was 30 minutes and the slip distance was 5 mm. In order to calculate the wear rate, the mass changes of each sample before and after the wear test were measured on an electronic analytical balance with an accuracy of 0.00001g. Three repeated tests were carried out and the average mass loss was taken as the final experimental value.

### 2.4. Microstructure Observation

The microstructures of the samples formed by the SLM were analyzed using an SU5000 scanning electron microscope (SEM) (Zeiss Ultra 55, Carl Zeiss Microscopy, Jena, Germany), which was equipped with an energy-dispersive X-ray spectral (EDS) analyzer. The samples were etched with Marble’s reagent for 10 s. In addition, transmission electron microscopy (TEM) investigations were conducted employing a JEOL 2010 TEM system (JEOL, Tokyo Metropolis, Japan). To clarify the wear mechanism, three-dimensional (3D) morphology and worn surface roughness were analyzed with a 3D laser scanning microscope (KEYENCE, Osaka, Japan, V-K-9700K). The cross-section perpendicular to the worn surface was prepared in order to discuss the microstructure evolution of subsurface further.

## 3. Results and Discussions

### 3.1. Microstructure of TiC/316Lss Composite

**Figure 2:**
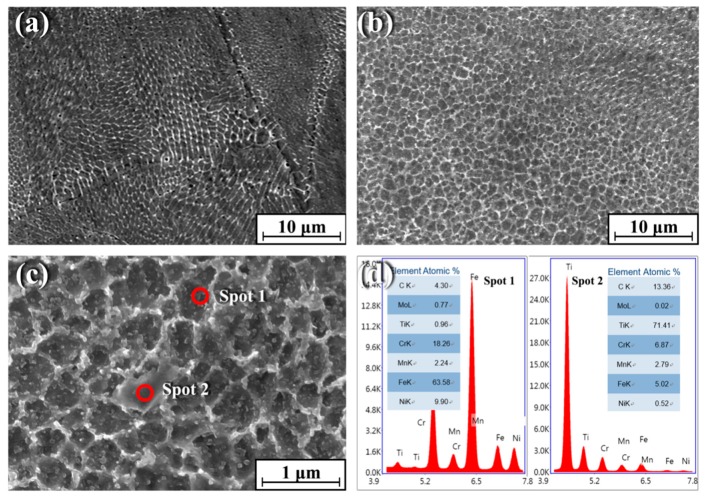
Scanning electron microscope (SEM) morphology of (**a**) the 316Lss matrix; (**b**) the TiC/316Lss composite; (**c**) high magnification SEM image of (**b**); (**d**) Energy dispersive X-ray spectral (EDS) spots analysis of (**c**).

Figure 2 shows the SEM microstructures of pure 316Lss (Figure 2a) and the TiC/316Lss composite (Figure 2b). According to Figure 2, the cells of 316Lss matrix are cellular and dendritic, while the cells of the TiC/316Lss composites show cellular dendritic morphology and the cell size of the TiC/316Lss composites are extremely fine. The average cell size of the 316Lss matrix is 0.58 μm and the TiC particles are uniformly distributed at the cell boundary of the 316Lss matrix, as shown in Figure 2c. The EDS spots analysis proves the existence of TiC (Figure 2d). The melting process of SLM is very fast and the degree of supercooling in the molten pool is very high, which can reach 10^6–7^K/s [19] and is conducive to the remarkable refinement of the cells of the 316Lss matrix and the TiC reinforced phase. This phenomenon indicates that the TiC particles can effectively refine cells, which is consistent with previous reports [20] that the TiC particles, as an effective heterogeneous nucleation point of primary cells, can lead to cell refinement of the 316Lss during solidification. In addition, the high forming temperature of SLM and intense Marangoni convection promote the wettability of the matrix and reinforced phase. Moreover, the laser dynamic pool with high energy has a great degree of supercooling, which is helpful for cell refinement, the non-equilibrium phase transformation and the supersaturated solid solution formation. At the same time, there are many kinds of fluids in the pool, which can promote the homogeneity of the structure. Therefore, the parts formed by SLM have good uniformity and comprehensive properties.

**Figure 3:**
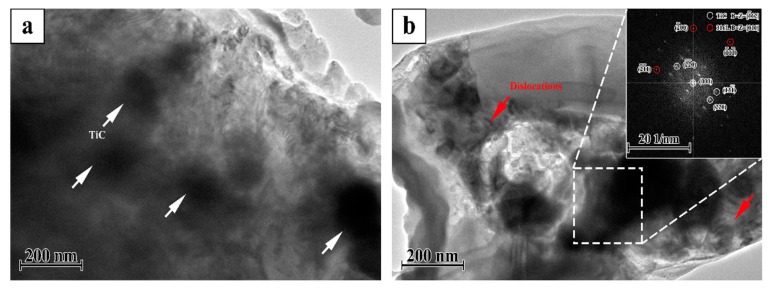
Transmission electron microscopy (TEM) micrographs (**a**,**b**) of the TiC/316Lss composite.

Figure 3 shows the TEM micrographs of the TiC/316Lss composites with 2 wt% TiC particles evenly distributed on the matrix. In addition, there are no impurities in the interface between the TiC reinforcement and the matrix. That is to say, in composite materials, the interface is very clean, the TiC particles bond well with the 316Lss matrix. Most importantly, as shown in Figure 3, a large number of dislocations were observed near the reinforcement particles, because the thermal expansion coefficient of the TiC (7.74 × 10^−6^/K) is quite different from that of the 316Lss (17.3 × 10^−6^/K). TiC particles promote heat dissipation in the molten pool [19,21], the increase of cooling and solidification speed leads to a decrease in the thermal expansion coefficient, which leads to high-density dislocations at the interface during the SLM.

### 3.2. Mechanical Properties

Table 1 shows the microhardness and compressive properties of the 316Lss and the TiC/316Lss composites. The results show that the microhardness, ultimate compressive strength (UCS) and compressive yield strength (CYS) of the TiC/316Lss composites are higher than those of unreinforced 316Lss. As shown in Figure 2a,b, the cell size of the composites reduces and the strength of the composites increases according to the fine cell strengthening and the TiC reinforcement will help to improve its bearing capacity [22]. The high density dislocation shown in Figure 3 also increases the strength of the TiC/316Lss composites. In addition, plastic deformation is more difficult due to the existence of residual dislocations during compression deformation, which improves the strength of composites.

### 3.3. Tribological Behavior of TiC/316Lss Composite

#### 3.3.1. Rate of Wear and Friction Coefficient

**Figure 4:**
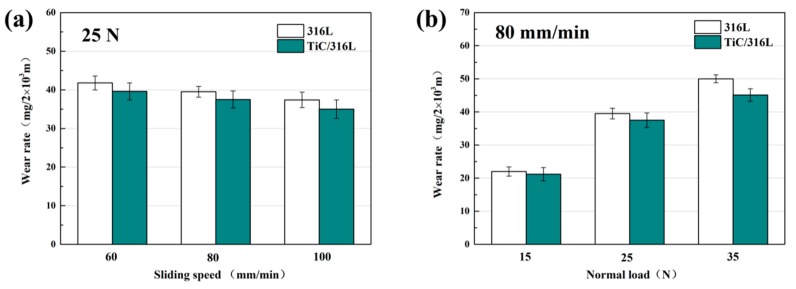
Wear rate of the matrix and composite: (**a**) at various sliding speeds under the load of 25 N; (**b**) under various load at the sliding speed of 80 mm/min.

Figure 4 shows the wear rate of 316Lss and the TiC/316Lss composites under the different sliding speeds and the loads of 15 N, 25 N and 35 N. The results show that the wear rate of the 316Lss and the TiC/316Lss composites decreases with the increasing of the sliding speed from 60 mm/min to 100 mm/min under the load of 25 N. When the sliding speed is 60 mm/min, the wear mechanism is abrasive wear and slight oxidation wear. When the sliding speed is increased to 100 mm/min, the wear mechanism changes to severe oxidation wear. The experimental results also show that the wear rates of 316Lss and composite materials are higher under larger loads at the sliding speed of 80 mm/min. In addition, when the load increases from 15 N to 35 N, the wear rate of the composites increases by nearly double, the fluctuation of the friction coefficient increases and the stability decreases. Similar trends in wear rates have been confirmed by previous studies [22]. In addition, it should be noted that the wear rate of the composite is lower than that of the matrix under all wear conditions.

The wear rate is calculated as follows:

(1) $$W^{\prime }=\frac{V}{PL}$$

In the above formulas, W′is the wear rate (mm3/Nm), V is the wear volume, P is the applied load (N), L is the sliding distance (m). Friction coefficient is measured by sensor and recorded by computer system. The formula for calculating wear volume is as follows:

(2) $$V=\frac{M_{loss}}{\rho }$$

Among them, Mloss is the weight change (g) of the sample before and after the experiment and *ρ* is the density (g/cm^3^) of the SLM formed sample.

That is to say, the composite with 2 wt% TiC reinforced particles has better wear performance, especially under a 35 N load. The results show that when the load is high, the wear rate of the unreinforced matrix and the reinforced composite is more different. Compared with the 316Lss matrix, mainly because of the increase in hardness and strength, the wear performance of the composite under a low load is improved [23,24,25]. In particular, the work hardening ability of the TiC/316Lss composites is improved due to the presence of the TiC particles under higher loads. Therefore, the hardness increases due to a higher degree of work hardening in composites, which will protect composites more effectively [26,27]. In previous studies, nanoceramic particles (TiC and TiB_2_) have been used as reinforcing materials in composite materials because of their excellent mechanical properties, and the wear resistance of the 316Lss matrix being improved and our experimental results are consistent with this.

**Figure 5:**
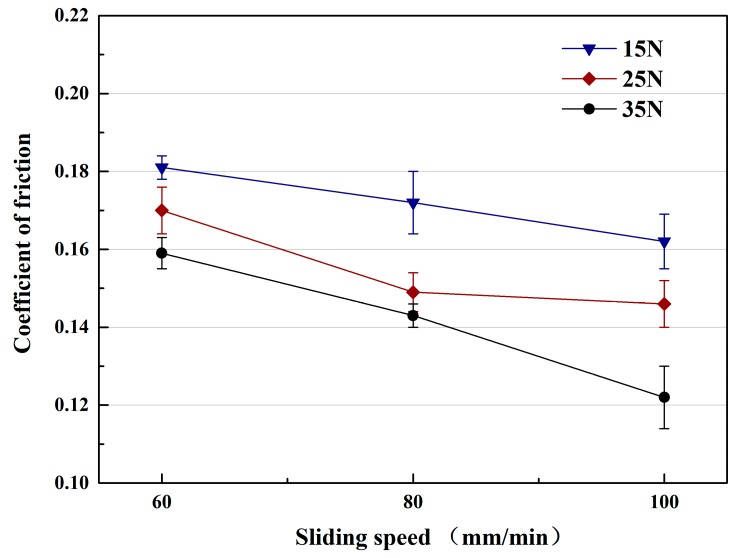
Coefficient of friction of the TiC/316Lss composite under various sliding speeds under the different loads of 15 N, 25 N and 35 N.

Figure 5 shows the friction coefficients (COF) of TiC/316Lss composites under the different loads of 15 N, 25 N and 35 N and the different sliding speeds, respectively. The friction coefficient (COF) under different loads also shows similar patterns to those at different sliding speeds, as shown in Figure 4. Under the same load, the friction coefficient decreases with an increase in sliding speed. At the same sliding speed, the friction coefficient also decreases with the increase of the load. However, in this case, the wear rate of the composites increases continuously with the increase of the load, which is contrary to the change of the coefficient of friction, which indicates that the change of the wear mechanism of the composite. Previous studies [28] have shown that the change is related to sliding speed and load. In order to illustrate the above results, the wear surface, debris and cross-section morphologies of 316Lss matrix and composites were analyzed.

#### 3.3.2. Tribological Behavior of Different Sliding Speeds

**Figure 6:**
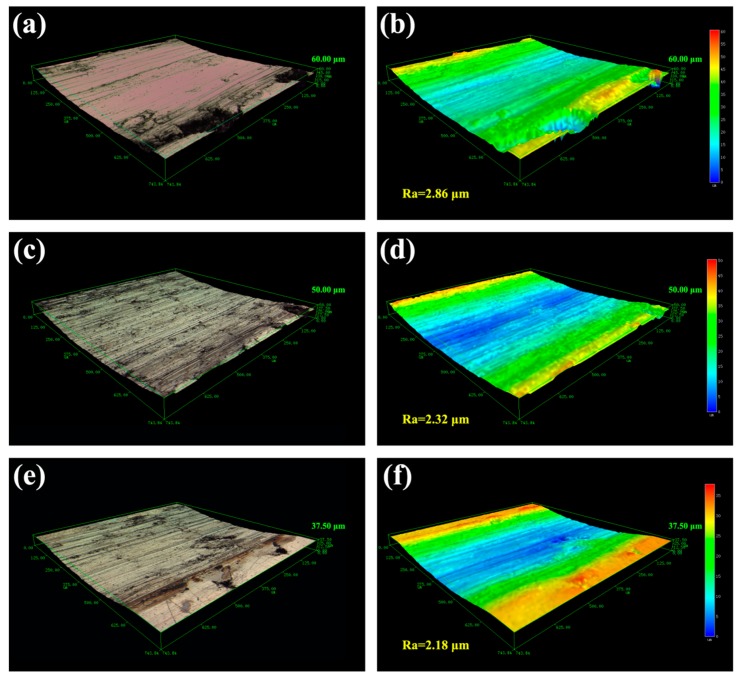
Macroscopic fracture morphology of worn surfaces for the TiC/316Lss composite at the load of 25 N at the sliding speed: (**a**) 60 mm/min; (**c**) 80 mm/min and (**e**) 100 mm/min; (**b**,**d**,**f**): 3D laser morphology and surface roughness corresponded to (**a**,**c**,**e**), respectively.

**Figure 7:**
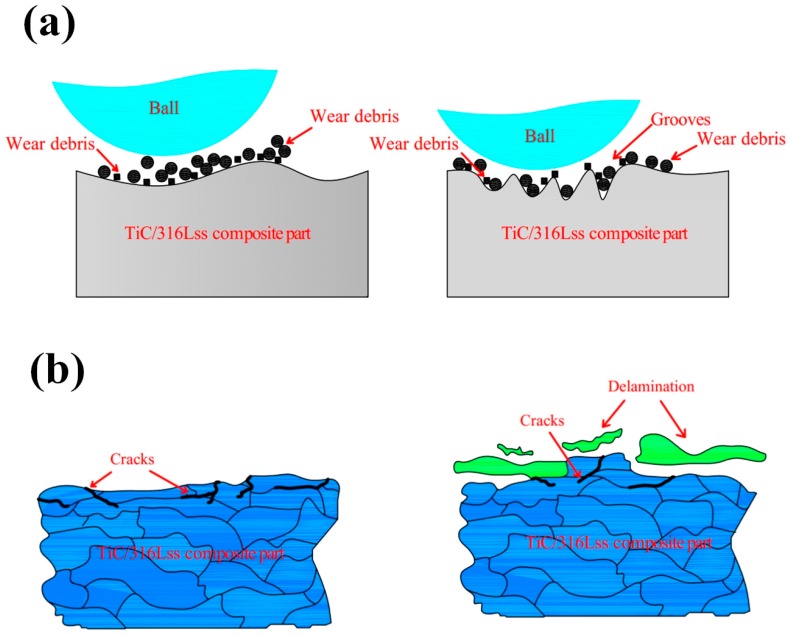
Schematic diagram of (**a**) abrasive wear mechanism; (**b**) delamination wear mechanism.

Figure 6 shows the macroscopic fracture morphology of the TiC/316Lss composites at the sliding speeds of 60, 80 and 100 mm/min under a load of 25 N. As shown in Figure 6a, around all the wear surfaces, at a lower sliding speed a number of furrows parallel to the sliding direction can be found and the wear deformation of the specimen surface is the most serious. The wear surface smoothness is greatly reduced, and the surface is rough. There are a large number of delamination cracks, large size pits and a large number of delamination blocks on the micro-wear surface [29,30]. In this case, the main wear mechanism is the severe delamination wear and these furrows are typical of abrasive wear [31], indicating that the primary mechanism of composite sliding at low speeds is abrasive wear. Figure 7a is a schematic diagram of the abrasive wear mechanism, which is a phenomenon of surface abrasion and material shedding caused by the relative movement of external hard particles or micro-peaks on the dual surfaces of friction pairs. As the sliding speed increases, some of the furrows on the wear surface become shallower and smaller and the wear damage becomes smaller as shown in Figure 6c. The surface plastic deformation and abrasive wear become shallower, the width and depth of scratches and furrows decrease greatly and the the delamination phenomenon is evidence on the worn surface and obvious delamination long cracks appear, which is in accordance with the reduction in wear rate as shown in Figure 4b. 

In addition, the morphology of the wear surface at the higher sliding speed of 100 mm/min that is shown in Figure 6e, which seems to be different from those at the lower sliding speed. A slight plastic deformation occurs on the wear surface of the specimen, resulting in shallow and narrow scratches and furrows along the sliding direction. High power SEM analysis shows that the scratches and furrow edges are scattered with uniformly dispersed fine debris, at which point the abrasive wear on the surface of the sample plays a dominant role. During the sliding wear process, most of the friction energy is converted into thermal energy (Q), it is written as follows:

(3) Q=μNΔx

where N is the acting force, Δx is the slip distance and μ is the friction coefficient. The heat generated makes the temperature of the friction and wear surface rise sharply. With the increase of velocity, more friction heat energy is generated. According to the above formula, the wear surface of composite materials is oxidized with increasing temperature. Therefore, this oxide layer will act as a protective layer to prevent contact between the disc and the composite surface [30]. In addition, as the friction speed increases, the wear rate of the composite material decreases, as shown in Figure 4a–c. This phenomenon is consistent with previous work and the resulting oxide layer protects the worn surface and reduces the amount of wear [31].

Slip speed is also an important factor affecting the tribological behavior of composites. In the experiment, the friction pair generates both wear and friction power on the surface of the sample. Most of the friction power is converted to friction heat, which increases the surface temperature of the sample. However, the temperature rise of the specimen surface varies with the sliding speed and the power of the friction pair. When the sliding speed is high, the more work the friction pair does per unit time, the more obvious the friction heat effect. With more heat storage on the worn surface, the higher the surface temperature rises. The movement of dislocations at the interface between the matrix and the TiC reinforcement phase is blocked and aggregated, which results in great stress concentration. With the slip, the dislocations continue to aggregate and pin, and the stress concentration continues to increase. When the interface bonding strength of the sample is lower than the stress, the TiC reinforcement phase will fall off from the interface and a large number of cracks will nucleate at the interface and gradually extend. In contrast, the roughness and depth of the worn surface decreases with the increase of the sliding speed. As shown in Figure 6d–f, a similar trend in wear rate and coefficient of friction (COF) is shown in Figure 4 and Figure 5. The maximum roughness (2.86 μm) and wear depth (60.0 μm) were obtained at the low sliding speed (60 mm/min), indicating that serious wear and material removal occurred on the worn surface. This is consistent with the results shown inf Figure 6a. In addition, as can be seen from Figure 6f, when the sliding speed is increased, the three-dimensional topography becomes smoother and the roughness is the lowest, which is a manifestation of slight wear. 

**Figure 8:**
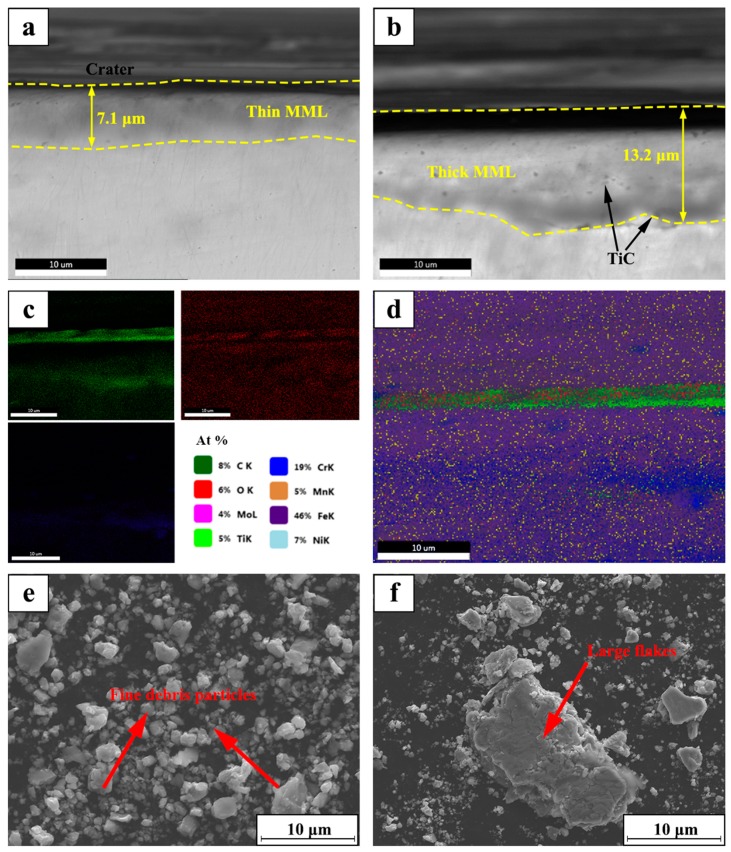
SEM micrograph of cross section for the TiC/316Lss composite under the load of 25 N at the speed: (**a**) 60 mm/min; (**b**) 100 mm/min; (**c**,**d**) EDX mapping of the O element and Fe element for (**b**); (**e**,**f**): SEM micrograph of wear debris corresponded to (**a**,**b**).

Figure 8 shows the SEM micrographs of the cross sections of the TiC/316Lss composites after friction and wear at the sliding speeds of 60 mm/min and 100 mm/min under a load of 25 N. For the observation of the sub-surface microstructures, as shown in Figure 8a, significant transition between the wear surface of the composite and the original tissue is presented. According to previous studies [17,30], the transition layer is a mechanically mixed layer (MML) of metal matrix composite formed under the dry sliding wear conditions. It is well known that the microhardness of a metal-based alloy composed of a work hardened layer and an oxide layer is 4-8 times higher than that of the substrate. Figure 8c shows the EDX mapping of O, Fe, Ti and C elements. In addition, it must be pointed out that the thickness of the mechanical mixed layer (MML) increases from 7.1 μm at the sliding speed of 60 mm/min to 13.2 μm at the sliding speed of 100 mm/min (Figure 8a,b). Therefore, the thick and stable mechanically mixed layer formed by the increase in frictional heat and the acceleration of the oxidation rate will reduce the wear rate by preventing direct contact with the steel friction pair at the high speed of 100 mm/min. It has been reported [32,33,34] that the formation of MML plays an important role in improving the abrasion performance of composites and our experimental results are consistent with this.

Figure 8e,f show the SEM morphology of the wear debris of the TiC/316Lss composites at the different sliding speeds of 60 mm/min and 100 mm/min under the load of 25 N. It has been found that, as the sliding speed increases, the morphology of the abrasive particles changes from fine to large sheets. As shown in Figure 8e, the fine debris particles produced by the third body hard abrasive micro-cutting on the worn surface are an indication of abrasive wear. However, as shown in Figure 8f, when the sliding speed is higher at 100 mm/min, the wear debris is a sheet-like morphology of about 10–20 μm in size, and the layered wear mechanism can explain this phenomenon [35]. Figure 7b is a schematic diagram of the delamination wear mechanism. According to Suh’s delamination theory [36], the friction pair produces repeated periodic compressive stress and shear stress by continuously grinding the surface of the specimen, which results in obvious plastic deformation on the surface of the specimen during the process of periodic friction and wear. Plastic deformation occurs not only on the surface of the specimen but also it extends to the sub-surface of the sample along the direction of the material stress field. The plastic deformation of the subsurface causes dislocations to accumulate and move, which leads to strain gradient. The strain gradient depends on the slip distance and the contact load and it increases with an increase of the slip distance and the contact load. Meanwhile, as shown in Figure 8b, the generation and expansion of composite structures and interface microcracks will result in the delamination of the metal matrix composite and large-scale plate debris, which further conforms to layered wear theory. It is worth mentioning that oxidative wear becomes the main wear mechanism with increasing sliding speed.

#### 3.3.3. Effect of Load on Friction and Wear Performance

**Figure 9:**
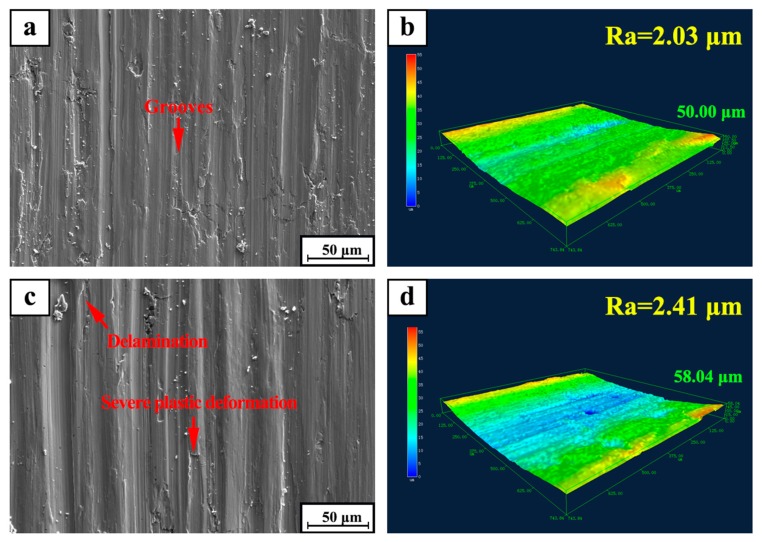
SEM micrograph of worn surfaces for TiC/316Lss composite under the sliding speed of 80 mm/min at different load: (**a**) 15 N; (**c**) 35 N; (**b**,**d**) 3D laser morphology and surface roughness of (**a**,**c**), respectively.

Figure 9 shows the SEM micrographs of the worn surfaces of TiC/316Lss composites at the sliding speeds of 80 mm/min under different loads (15 N and 35 N). It can be seen that the wear morphology of the samples varies greatly with different external loadings. As shown in Figure 9a, it can be seen that when the external load is lower than 15 N, the wear degree of the surface of specimen is lighter. At the same time, the wear surface is flat and smooth without obvious damage and defects and the surface damage is not serious. When the load is up to 35 N, the worn surface is seriously damaged. At this time, the surface is composed of a series of parallel furrows with a deeper depth and wider width. There is a large amount of large-scale debris on the edge of the furrow. As shown in Figure 9c, it shows that severe deformation and ploughing take place on the surface. At the same time, slight delamination occurs on the surface of the specimen. Some irregular delamination occurs on the wear surface of the specimen, this is consistent with an increase in wear rate and an increase in load as shown in Figure 5. Based on the above analysis of wear surfaces, it can be inferred that plastic deformation and delamination are the main mechanisms. Figure 9b,d show that the three-dimensional laser morphology and surface roughness of the worn surface of the composites after friction and wear at a speed of 80 mm/min under the loads of 15 N and 35 N. The results show that the surface roughness depends to a large extent on the degree of wear damage. Severe delamination or wear damage will result in greater surface roughness values, which is consistent with results at higher loads of 35 N.

**Figure 10:**
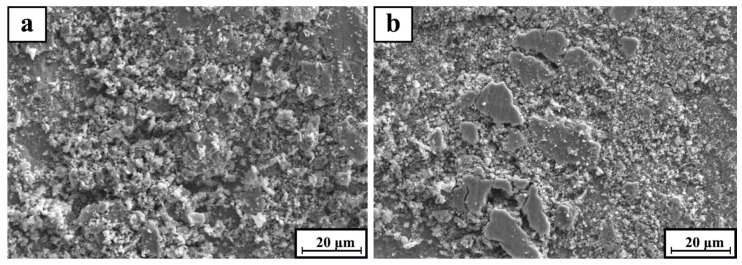
SEM micrograph of wear debris for TiC/316Lss composite at the sliding speed of 80 mm/min at the different load: (**a**) 15 N and (**b**) 35 N.

Figure 10a,b show that the SEM morphology of the wear particles of TiC/316Lss composites at different loads of 15 N and 35 N and the sliding speeds of 80 mm/min. Fine abrasive particles appear at lower loads, indicating that the wear mechanism is mainly abrasive wear. However, under the load of 35 N, the fragments are in the shape of a large metal plate of 10 μm × 20 μm. As shown in Figure 10b, as the load increases, delamination wear becomes the primary wear mechanism. The crumbs have similarities in size and shape under the different loads and the different sliding speeds. However, due to the large shear strain, the delamination of the mechanical mixed layer (MML) or subsurface is more severe at high loads.

#### 3.3.4. Effect of TiC Particle Addition on Friction and Wear Properties

**Figure 11:**
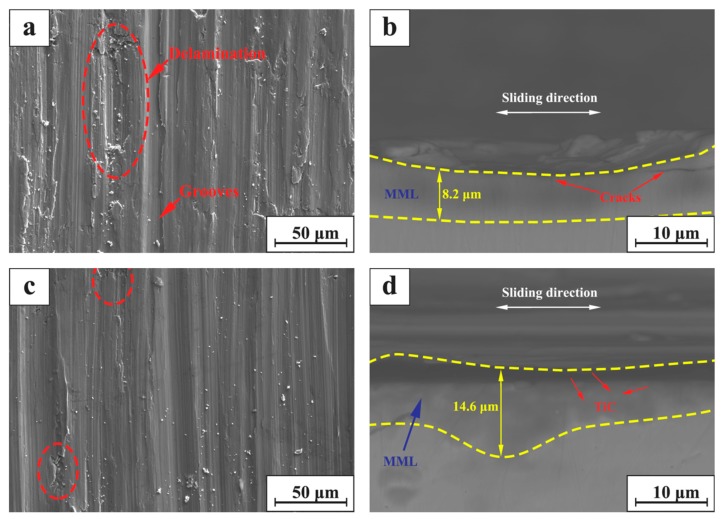
SEM micrograph of worn surface for (**a**) the TiC/316Lss composite and (**c**) the 316Lss matrix; SEM micrograph of cross section for (**b**) the 316Lss matrix and (**d**) the TiC/316Lss composite at the sliding speed of 100 mm/min at the load of 35 N.

Figure 11a,c show the SEM micrographs of the surfaces of 316Lss and TiC/316Lss composite at 100 mm/min under the load of 35 N, respectively. Obviously, the wear damage of the 316Lss matrix (Figure 11a, such as delamination, pit, groove and material removal, is much more serious than composite materials as shown in Figure 11c. The results show that the wear properties of the composites containing TiC reinforcing particles are higher than 316Lss under all conditions. Figure 11b,d show the SEM diagrams of vertical sections of 316Lss and TiC/316Lss composites at the sliding speed of 100 mm/min under the load of 35 N, respectively. It is easy to see that there are a lot of cracks in the MML of 316Lss as shown in Figure 11b, which indicates that the strength of 316Lss is relatively low. Therefore, during friction and wear, these cracks gradually expand and cause delamination of the mechanical mixed layer or subsurface.

**Figure 12:**
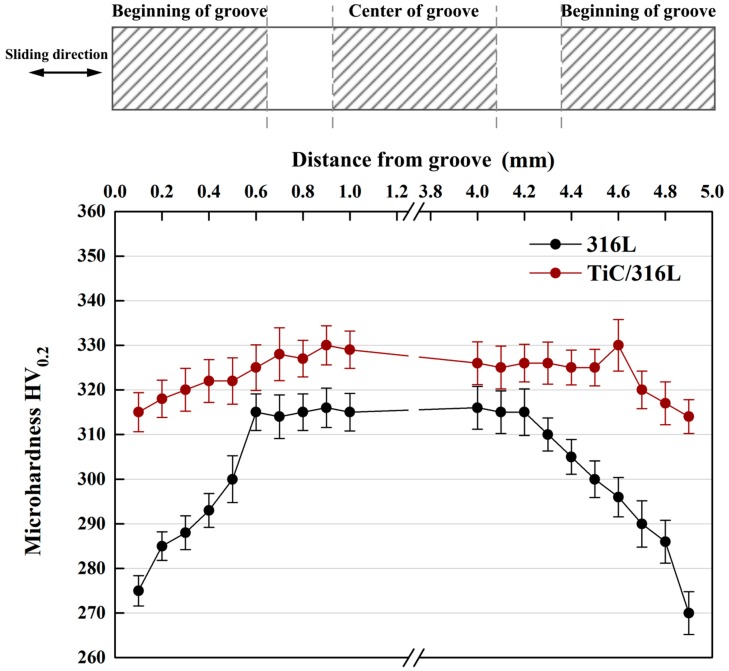
Micro-hardness of subsurface layers for the matrix and composite after sliding wear test (sliding speed: 100 mm/min and the load: 35 N).

According to the report [37,38], the work hardening of the subsurface layer is obvious under the high cyclic load and sliding speed. For the sake of explaining the effect of TiC particles on the reinforcement of composites, Figure 12 shows the micro-hardness of 316Lss and TiC/316Lss composites after sliding wear test when the sliding speed is 100 mm/min and the load is 35 N. The results show that the micro-hardness of the composite is obviously higher than that of the matrix alloy. In addition, it is worth noting that, compared with the unreinforced 316Lss, the microhardness of MML in the middle part of the wear of composites is significantly increased, while at the beginning and the end of the wear, the hardness is lower than that in the middle because of large plastic deformation.

In other words, the work hardening ability of 316Lss matrix composites containing the TiC particles is significantly improved. The presence of the TiC particles results in the high barrier effect of dislocation movement during deformation. Therefore, the high work hardening rate makes the sub-surface of composites have high hardness and strength, which is sufficient to support the bottom surface, helping to improve wear resistance. Meanwhile, there are no cracks in the MML of the composite (Figure 11b,d). The thickness of 14.6 μm MML is larger than that of 8.2 μm in 316Lss matrix, which confirms the above results.

In addition, with the gradual increase of the sliding speed and load, the work hardening rate and micro-hardness of the composites increases, which can protect the metal matrix composites and help to reduce delamination wear and improve abrasion performance.

## 4. Conclusions

This study has investigated the effect of TiC particles on the abrasion performance of composite in various dry sliding conditions. The morphology evolution of worn surface and subsurface are analyzed. The main research results can be summarized as follows:

(1) The addition of TiC particles can effectively refine the cell size of the TiC/316Lss composite. The results of TEM show that the reinforcement material is well bonded with the 316Lss and there are a large number of dislocations near the TiC particles, which significantly improves the micro-hardness and strength of the composite.

(2) The wear rate of composites decreases with the increase of the sliding speed. When the sliding speed is 100 mm/min, the wear mechanism is abrasive wear and slight oxidation wear. When the sliding speed is increased to 60 mm/min, the wear mechanism changes to severe oxidation wear. In addition, when the load increases from 15 N to 35 N, the wear rate of the composites increases by nearly double, the fluctuation of the friction coefficient increases and the stability decreases. 

(3) Under all dry sliding conditions, the abrasion performance of the composites has been significantly improved by adding TiC particles. This is because the higher work hardening rate improves the hardness of the composites, which can support the surface of the composites more effectively.

## Figures and Tables

**Table 1:** The mechanical properties of the matrix and TiC/316Lss composite.

Materials	Hardness (HV0.2)	CYS (MPa)	UCS (MPa)	Failure Strain (%)
316Lss	298 ± 22	171 ± 7	482 ± 12	23.2 ± 0.3
TiC/316Lss	335 ± 8	185 ± 8	505 ± 9	24.8 ± 0.5
